# Early prediction of acquiring acute kidney injury for older inpatients using most effective laboratory test results

**DOI:** 10.1186/s12911-020-1050-2

**Published:** 2020-02-20

**Authors:** Yi-Shian Chen, Che-Yi Chou, Arbee L.P. Chen

**Affiliations:** 1 Department of Computer Science, National Tsing Hua University, Hsinchu, Taiwan; 2 Division of Nephrology, Asia University Hospital, Taichung, Taiwan; 3 Department of Post-baccalaureate Veterinary Medicine, Asia University, Taichung, Taiwan; 4 Kidney Institute and Division of Nephrology, China Medical University Hospital, Taichung, Taiwan; 5 Department of Computer Science and Information Engineering, Asia University, Taichung, Taiwan; 6 Department of Medical Research, China Medical University Hospital, China Medical University, Taichung, Taiwan

**Keywords:** Acute kidney injury, Early prediction, Electronic health records, Laboratory tests, K nearest neighbors

## Abstract

**Background:**

Acute Kidney Injury (AKI) is common among inpatients. Severe AKI increases all-cause mortality especially in critically ill patients. Older patients are more at risk of AKI because of the declined renal function, increased comorbidities, aggressive medical treatments, and nephrotoxic drugs. Early prediction of AKI for older inpatients is therefore crucial.

**Methods:**

We use 80 different laboratory tests from the electronic health records and two types of representations for each laboratory test, that is, we consider 160 (laboratory test, type) pairs one by one to do the prediction. By proposing new similarity measures and employing the classification technique of the K nearest neighbors, we are able to identify the most effective (laboratory test, type) pairs for the prediction. Furthermore, in order to know how early and accurately can AKI be predicted to make our method clinically useful, we evaluate the prediction performance of up to 5 days prior to the AKI event.

**Results:**

We compare our method with two existing works and it shows our method outperforms the others. In addition, we implemented an existing method using our dataset, which also shows our method has a better performance. The most effective (laboratory test, type) pairs found for different prediction times are slightly different. However, Blood Urea Nitrogen (BUN) is found the most effective (laboratory test, type) pair for most prediction times.

**Conclusion:**

Our study is first to consider the last value and the trend of the sequence for each laboratory test. In addition, we define the exclusion criteria to identify the inpatients who develop AKI during hospitalization and we set the length of the data collection window to ensure the laboratory data we collect is close to the AKI time. Furthermore, we individually select the most effective (laboratory test, type) pairs to do the prediction for different days of early prediction. In the future, we will extend this approach and develop a system for early prediction of major diseases to help better disease management for inpatients.

## Background

Acute Kidney Injury (AKI) affects 5–7% of inpatients [1] and 22–57% of patients in the intensive care unit [2–6]. Severe AKI can result in significant mortality (40–70%) for critically ill patients [7]. Older inpatients have the highest incidence of AKI due to multiple contributing factors [8–11], including increased number of comorbidities, aggressive medical treatments, and greater use of nephrotoxic drugs [12–15]. Even after resolution of AKI, it can subsequently lead to severe kidney problems such as progression to dialysis dependency and chronic kidney disease [1, 16]. Therefore, early recognizing high-risk older inpatients to prevent them from acquiring AKI is important for physicians. It can give physicians adequate time to modify practice, and help better disease management for the patients. In this study, we propose an approach to early predict AKI up to 5 days prior to possible AKI events for the inpatients over age 60.

AKI is defined using Acute Kidney Injury Network (AKIN) criteria [7] which is based on the serum creatinine or urine output. However, the AKIN criteria can only identify AKI after the inpatients have already acquired AKI. The Acute Dialysis Quality Initiative (ADQI) Consensus Conference [17] recommended that forecasting AKI events with a horizon of 48 to 72 h would give physicians adequate time to modify practice. In this study we develop an approach to early predict AKI up to 5 days prior to the possible AKI event.

In recent years, the application of computer technologies to medicine has become a hot research field, and among the technologies machine learning is most adopted. It identifies patterns of diseases using patient electronic health records (EHR) and informs physicians of any anomalies. To predict AKI for inpatients by machine learning, Kate et al. [18] built models to predict AKI for older inpatients at 24 h of admission. In addition, Cheng et al. [19] built models to predict AKI 0 to 5 days prior to the possible AKI event for inpatients aged 18–64. Mohamadlou et al. [20] built models to early detect and predict AKI for the inpatiens older than age 18. Nenad et al. [21] built a model for the continuous risk prediction of future deterioration inpatients.

In order to early predict AKI, we use a classification method. A patient is classified to a potential AKI patient if most of the patients with similar features based on EHRs are AKI patients. On the other hand, a patient is classified to a non-AKI one if most of the patients with similar EHR features are non-AKI patients. In this study, we use the laboratory test data and the corresponding similarity measures to determine the similarity of two patients. For a laboratory test, an inpatient may have several values from different times of the test. A sequence of the laboratory test values can therefore be formed. Two types of data from the sequence are then extracted to represent this sequence. The first type is the *last value* in the sequence, and the second type is the *trend* of the sequence. The trend of the sequence represents the rate of increase or decline of the values. In order to avoid minor differences, we transform the last value and the trend of the sequence into symbolic values for further processing.

We then employ two different similarity measures for the last value and the trend of the sequence. For the last value, we compute the difference of the values as the similarity score of each pair of patients. For the trend of the sequence, we use a similarity measure to obtain the similarity score of each pair of patients because the lengths of the individual sequences are often different. Finally, we consider 80 different laboratory tests from the EHR and the two types of sequence representations for each laboratory test, that is, we consider 160 (laboratory test, type) pairs one by one to classify the patients. Since not all of these (laboratory test, type) pairs can help for the AKI prediction, we use two selection methods, i.e. sequential forward selection (SFS) [22] and sequential backward selection (SBS) [23] to select the most effective (laboratory test, type) pairs.

**Fig. 1:**
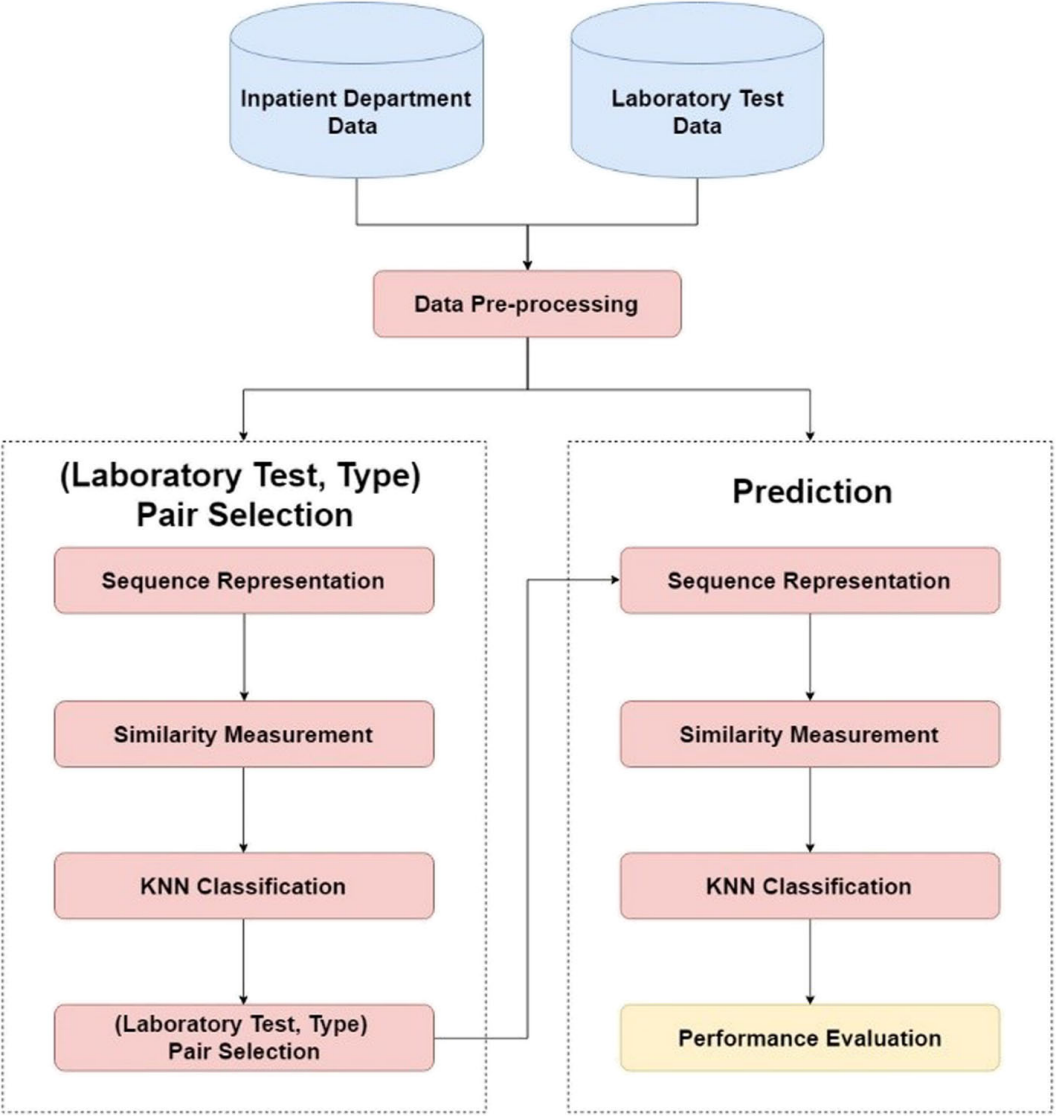
The framework of our AKI prediction approach

The framework of our AKI prediction method is shown in Fig. 1. We first preprocess the inpatient department data and laboratory test data to obtain the dataset for further processing. Then, the dataset is divided into two parts, one for selecting the most effective (laboratory test, type) pairs and the other for doing prediction. To select the most effective (laboratory test, type) pairs, we first transform the last value and the trend of the sequences into symbolic values. Then, we employ the similarity measurements and the classification method to obtain the result for each (laboratory test, type) pair, and select the most effective (laboratory test, type) pairs. In the prediction part, we use these effective (laboratory test, type) pairs to do the prediction. Similarly, we transform the last value and the trend of the sequences into symbolic values for the (laboratory test, type) pairs we selected in the first part. Finally, we employ the similarity measurements and the classification method to obtain the prediction result, and evaluate the performance.

## Methods

### Description of the dataset

The dataset is from China Medical University Hospital which contains the inpatient department data, outpatient department data, emergency department data, medication data, and laboratory test data from 2003 to 2015. In this study, we use the inpatient department data and laboratory test data. There are 561,222 encounters in the inpatient department data. The inpatient department data includes the inpatient ID, admission date, discharge date, admission diagnosis, and discharge diagnosis. The laboratory test data includes the inpatient ID, laboratory test code, laboratory value, and laboratory time. We link the inpatient department data and laboratory test data based on the inpatient ID. An inpatient may have multiple admissions (*encounters*). In some admissions the AKI might occur, and in some others it might not. We define the exclusion criteria to identify the inpatients who develop AKI in a specific admission, i.e. it ensures the inpatients did not acquire AKI when they were admitted to the hospital, and developed AKI during the hospitalization. Therefore, we treat each admission individually and use the corresponding laboratory tests to do the prediction. In addition, we need to ensure the laboratory time is within the admission time and discharge time for an encounter. A sequence of the laboratory test values for a laboratory test can therefore be formed for an encounter. Notice that the number of laboratory tests varies from patient to patient.

### Preprocessing of the dataset

AKI is defined using AKIN criteria [7] which is based on the serum creatinine or urine output. Although the urine output is one of the diagnostic criteria of AKI, it can be influenced by factors other than renal health. In this study, we use the serum creatinine to distinguish the AKI inpatients and non-AKI inpatients based on the following criterion: A patient with an absolute increase in serum creatinine of 0.3 mg/dL or increase to 150–200% within 48 h is classified as “AKIN stage 1.” The inpatients classified to AKI inpatients need to meet the AKIN stage 1 criteria. In addition, we strictly extract the inpatients who develop AKI during hospitalization to make the results more convincing. That is, before the inpatients acquire AKI, we ensure that there is one pair of serum creatinine measurements taken within 48 h and the increase of the second serum creatinine measurement does not exceed the threshold defined in the AKIN criteria. For the non-AKI inpatients, we ensure that there is at least one pair of serum creatinine measurements taken within 48 h and all pairs of serum creatinine measurements do not meet the AKIN criteria. Furthermore, the laboratory test data before the *AKI Time* (the laboratory time of the second serum creatinine measurement which first meets the AKIN criteria) was collected for the AKI inpatients. For non-AKI inpatients, we collect the laboratory test value before the laboratory time of the last serum creatinine measurement.

**Fig. 2:**
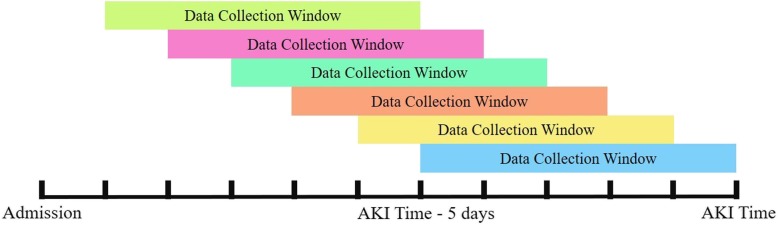
An illustration of adjusting the prediction time

We evaluate the prediction performance at 0 to 5 days prior to the AKI event. The data collection window is denoted as [lower_bound, upper_bound]. We set the length of data collection window to 5 days and adjust the prediction time as shown in Fig. 2. To illustrate with an example, an inpatient acquires AKI at 2010-08-25 14:00 (the AKI Time), then the data collection window for predicting at 2-days prior to the AKI event would be [AKI Time – 7 days, AKI Time – 2 days] which is [2010-08-18 14:00, 2010-08-23 14:00]. In order to evaluate the performance of different prediction times, we collected data from the inpatients who stayed at least 10 days from admission to the AKI time. The data collected include 5 days for the prediction and 5 days for evaluating the performance of the prediction times. In order to relax the requirement of the 5 days data collection window for the prediction, we also consider other lengths of the data collection window, i.e. 1 day and 3 days, and evaluate the prediction performance accordingly.

**Fig. 3:**
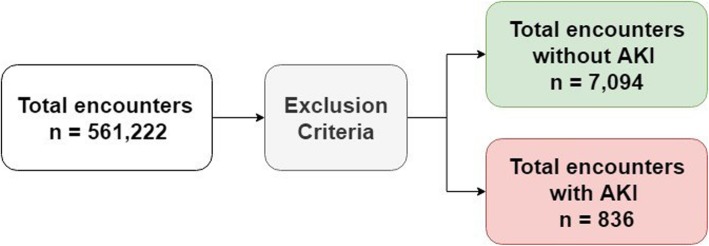
The number of AKI encounters and non-AKI encounters in the dataset

In addition, we exclude the inpatients younger than 60 years of age since we focus on the older inpatients in this study. Out of the total of 7930 encounters included in our data, 836 (10.54%) encounters acquire AKI as shown in Fig. 3.

**Fig. 4:**
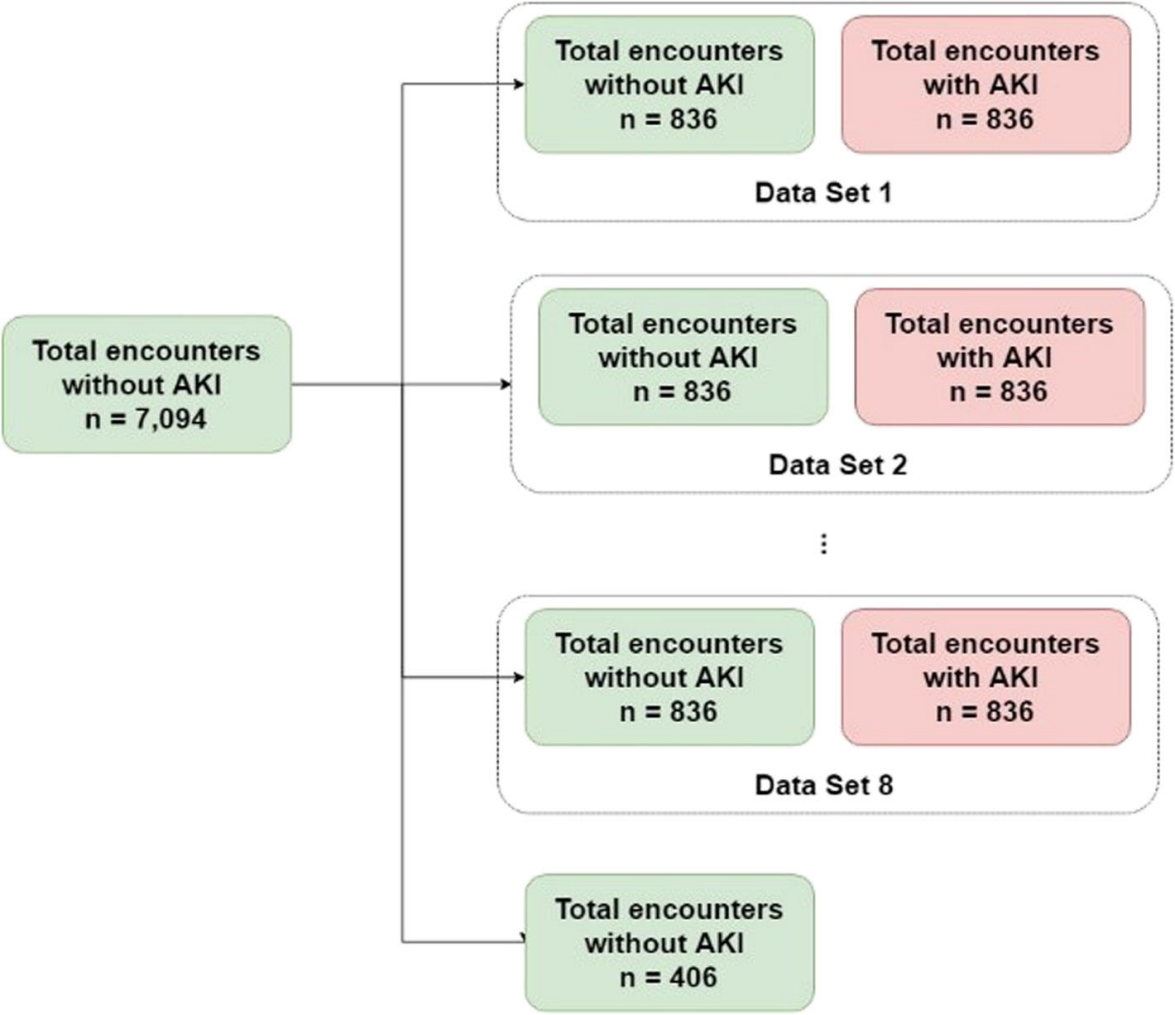
The number of encounters in each data set

Our dataset is highly imbalanced with an approximate 1:8 ratio for the AKI inpatients to non-AKI inpatients. With such an imbalanced dataset, most classifiers will favor the majority class (non-AKI inpatients), resulting in a poor accuracy in the minority class (AKI inpatients) prediction. In this study, we randomly under-sampled the non-AKI inpatients such that the ratio for the AKI inpatients to non-AKI inpatients is 1:1. We repeatedly and randomly under-sampled the remaining non-AKI inpatients until the remaining non-AKI inpatients are less than the AKI inpatients. That is, we generated 8 data sets as shown in Fig. 4. We also compare the performance of using the imbalanced data and balanced data.

**Fig. 5:**
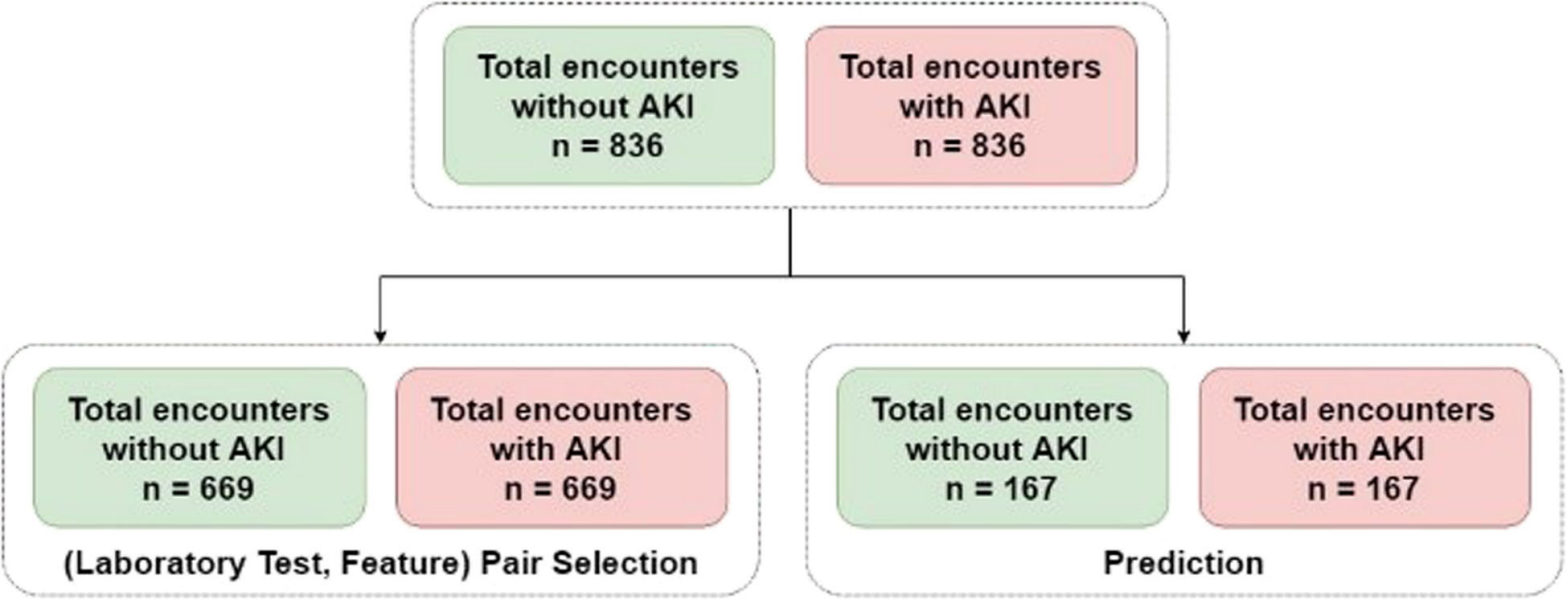
The number of encounters in (laboratory test, feature) pairs selection part and prediction part

Finally, we divide each data sets into two parts, one for selecting the (laboratory test, type) pairs and the other for doing prediction. We consider two ratios for these two parts, i.e. 8:2 and 5:5. Figure 5. Shows the number of encounters in these two parts for the case of the 8:2 ratio. Out of a total of 1338 encounters that are included in (laboratory test, type) pairs selection, and 334 encounters that are included in prediction. The AKI sample to non-AKI sample ratio is 1:1 in both (laboratory test, type) pairs selection and prediction.

### Segmentation and data representation

The sequence of the laboratory test values for an inpatient is denoted L = ((*t*_1_, *v*_1_), (*t*_2_, *v*_2_), …(*t*_*n*_, *v*_*n*_)) where *t*_1_ is the first laboratory time, and *v*_1_ is the first laboratory value of the inpatient, and *t*_*n*_ is the last laboratory time, and *v*_*n*_ is the last laboratory value. We extract two types of data from the sequence of the laboratory test values to represent the sequence. The first type of data is the *last* value of the sequence, denoted *v*_*last*_, *v*_*last*_ = *v*_*n*_.

In order to avoid minor differences, we transform the last value *v*_*last*_ into a symbolic value *r*_*last*_ according to the following formula, where *μ*_*last*_ denotes the mean and *σ*_*last*_ denotes the standard deviation of *v*_*last*_ for all inpatients. It can transform similar values to the same symbolic value.

$$ {r}_{last}=\Big\{{\displaystyle \begin{array}{ll}1& {v}_{last}\le {\mu}_{last}-{\sigma}_{last}\\ {}2& {\mu}_{last}-{\sigma}_{last}<{v}_{last}\le {\mu}_{last}-\frac{\sigma_{last}}{2}\\ {}3& {\mu}_{last}-\frac{\sigma_{last}}{2}<{v}_{last}\le {\mu}_{last}\\ {}4& {\mu}_{last}<{v}_{last}\le {\mu}_{last}+\frac{\sigma_{last}}{2}\\ {}5& {\mu}_{last}+\frac{\sigma_{last}}{2}<{v}_{last}\le {\mu}_{last}+{\sigma}_{last}\\ {}6& {\mu}_{last}+{\sigma}_{last}<{v}_{last}\end{array}}\operatorname{} $$

The second type of data is the trend of the sequence. Similarly, we transform the sequence of the laboratory test values into the *slope* sequence S = (*s*_1_, *s*_2_, …, *s*_*n* − 1_), where *s*_*j*_ is the slope between two adjacent laboratory values,

$$ {s}_j=\frac{v_{j+1}-{v}_j}{t_{j+1}-{t}_j} $$

We calculate the difference of time in minutes and convert it into days. For example, the sequence of the laboratory test values of an inpatient is ((2004-06-05 07:38, 88), (2004-06-09 17:22, 104), (2004-06-12 22:13, 137)). The difference of 2004-06-05 07:38 and 2004-06-09 17:22 is 4.41 days. The slope sequence S = (3.63, 10.31). The longer the time interval between the two laboratory tests is, the smaller the slope will be. Then, we transform the slope sequence into a symbolic slope sequence *R* = (*r*_1_, *r*_2_, …, *r*_*n* − 1_) according to the following formula, where *r*_*j*_ is the symbolic representation of *s*_*j*_, *μ*_*slope*_ denotes the mean, and *σ*_*slope*_ denotes the standard deviation of *s*_*j*_ ’s in the slope sequence S for all inpatients.

$$ {r}_j=\Big\{{\displaystyle \begin{array}{ll}1& {s}_j\le {\mu}_{slope}-{\sigma}_{slope}\\ {}2& {\mu}_{slope}-{\sigma}_{slope}<{s}_j\le {\mu}_{slope}-\frac{\sigma_{slope}}{2}\\ {}3& {\mu}_{slope}-\frac{\sigma_{slope}}{2}<{s}_j\le {\mu}_{slope}\\ {}4& {\mu}_{slope}<{s}_j\le {\mu}_{slope}+\frac{\sigma_{slope}}{2}\\ {}5& {\mu}_{slope}+\frac{\sigma_{slope}}{2}<{s}_j\le {\mu}_{slope}+{\sigma}_{slope}\\ {}6& {\mu}_{slope}+{\sigma}_{slope}<{s}_j\end{array}}\operatorname{} $$

### Similarity measures

We employ two different similarity measures for the last value and the trend of the sequence. The similarity score for the last value (*LS*) is measured by the difference of the symbolic values between each pair of target inpatient *r*_*last* _ *target*_ and the other inpatient *r*_*last* _ *other*_, i.e.

$$ LS=\mid {r}_{last\_ target}-{r}_{last\_ other}\mid $$

where the smaller the *LS* is, the more similar the two inpatients are. If the difference is zero, it means the symbolic values are the same, and the two inpatients are most similar. For the trend of the sequence, the trend similarity score (*TS*) is obtained by Dynamic Time Warping (DTW) [24]. DTW is a classic similarity measure. It has been widely applied to measure the similarity for two sequences. A well-known application is the automatic speech recognition which matches a sample voice with another voice with a faster or slower pace than the sample voice. It executes a mapping of one sequence to the other and find the best mapping with the minimum distance. Given two symbolic slope sequences ${R}_1=\left({r}_1^1,{r}_2^1,\dots, {r}_m^1\right)$ and ${R}_2=\left({r}_1^2,{r}_2^2,\dots, {r}_n^2\right)$, there is an m by n matrix *D* with elements *D*(*i*, *j*). The distance function *δ* between *R*_1_ and *R*_2_ is defined as $\delta \left(i,j\right)=\left|{r}_i^1-{r}_j^2\right|$. The matrix *D* can be constructed with the initial condition *D*(1, 1) = *δ*(1, 1). The matrix is filled in one element at a time following a column-by-column or row-by-row order. The element *D*(*i*, *j*) which denotes the cumulative distance is determined by the recursive formula below.

$$ D\left(i,j\right)=\delta \left(i,j\right)+\mathit{\min}\left\{D\left(i,j-1\right),D\left(i-1,j-1\right),D\left(i-1,j\right)\right\} $$

**Fig. 6:**
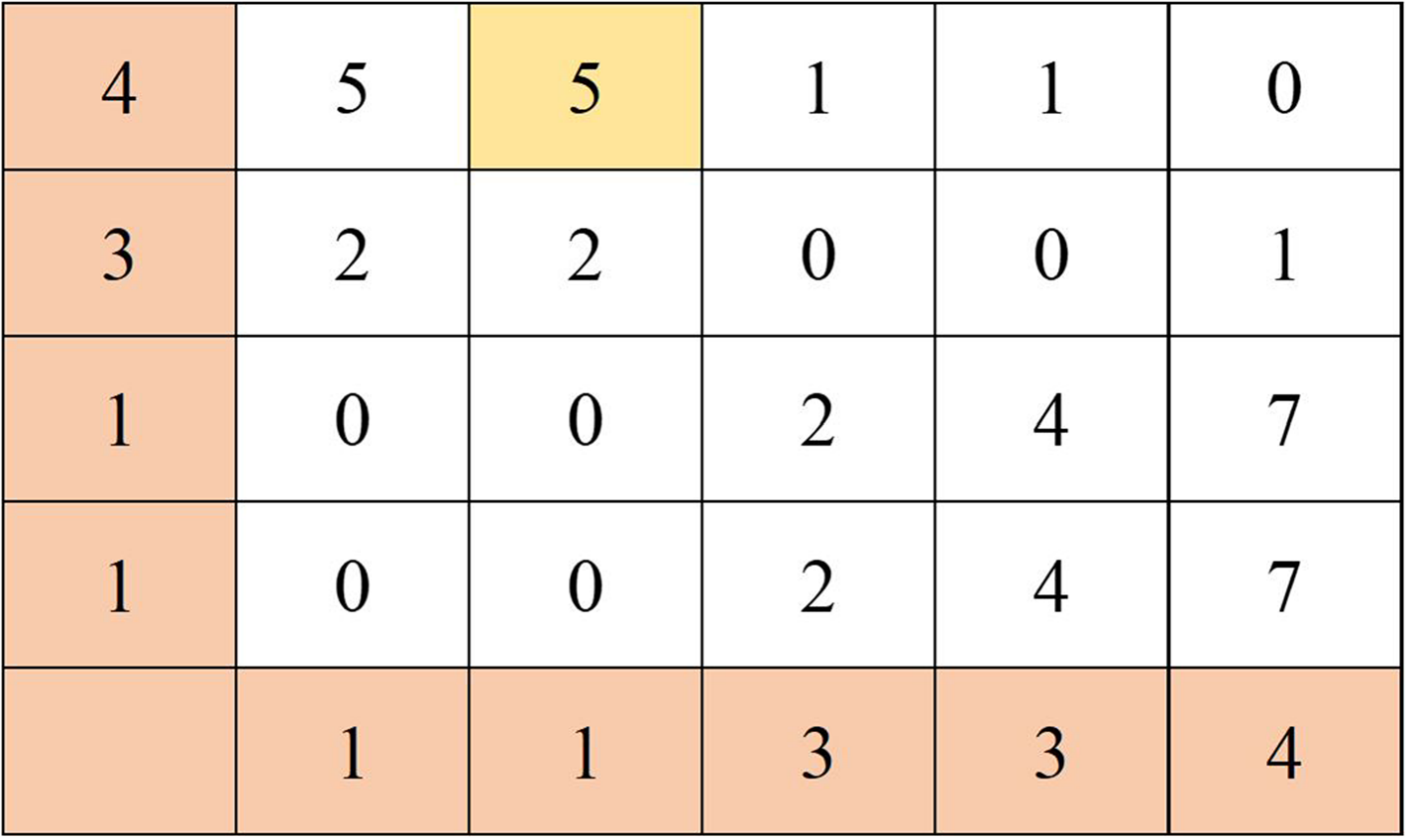
Computing the minimum distance of (1,1,3,3,4) and (1,1,3,4) by DTW

The resultant *D*(*m*, *n*) is the minimum distance between the two sequences. For the example shown in Fig. 6, sequence *S* = (1, 1, 3, 3, 4) and sequence *T* = (1, 1, 3, 4), m = 5 and n = 4. The distance function *δ* is defined as |*s*_*i*_ − *t*_*j*_|. The matrix *D* (5,4) can be constructed as follows. The initial element *D*(1, 1) is the distance of *s*_1_ and *t*_1_, which is 0. We fill in the element row-by-row. The element *D*(2, 1) is the sum of the distance between *s*_2_ *and t*_1_ and *D*(1, 1), resulting in 0. The element *D*(2, 4) is 5 as the sum of the distance between *s*_2_ and *t*_4_ (which is 3) and the minimum of the cumulative distances of *D*(1, 4), *D*(1, 3), and *D*(2, 3) (which is 2). Finally, *D*(5, 4) = 0 is the minimum distance between the two sequences.

### K-nearest neighbor classification

We use the K-nearest neighbor (KNN) classification method [25] to determine whether the target inpatient is an AKI inpatient. The idea behind KNN classification is that similar data points should have the same class. The principle is classifying a data point based on how its K neighbors are classified. We calculate the distance between the target point to be classified and all other points. The resultant distances are then ranked to find the K nearest neighbors. The majority class of the K nearest neighbors is designated as the class of the target point. In this study, the distance is used to measure the similarity score between each pair of inpatients. The smaller the distance is, the more similar the two inpatients are. For a laboratory test, there are two different similarity scores, i.e. the *LS* and *TS*. Therefore, we consider 160 (laboratory test, type) pairs one by one to classify the patients. Since not all of these (laboratory test, type) pairs can help for the AKI prediction, we select the most effective (laboratory test, type) pairs.

### (laboratory test, type) pair selection

In this study, we use the SFS [22] and SBS [23] strategies to select the most effective (laboratory test, type) pairs. For an inpatient, if there exist one (laboratory test, type) pair which classifies the inpatient to be positive, we classify the inpatient to be positive. Then, we use the SFS and SBS strategies to select the most effective (laboratory test, type) pairs by the accuracy of the different combinations of the (laboratory test, type) pairs. For SFS, we start with an empty set. With each iteration, one (laboratory test, type) pair among the remaining (laboratory test, type) pairs is added to the subset so that the subset maximizes the evaluation performance. For SBS, we exclude each (laboratory test, type) pair to compute the accuracy. We remove the (laboratory test, type) pair if the accuracy is the maximum when we exclude the (laboratory test, type) pair. We keep removing the (laboratory test, type) pairs until there is only one remaining (laboratory test, type) pair. Finally, we find the most effective (laboratory test, type) pairs to do the prediction.

### Prediction by the most effective (laboratory test, type) pairs

We repeat the process in the (laboratory test, type) pair selection part for the prediction part and use the selected most effective (laboratory test, type) pairs to do the prediction. Finally, we classify the inpatient to be positive if there exist one (laboratory test, type) pair which classify the inpatient to be positive.

## Results

We first compare our method with Kate et al. [18] and Cheng et al. [19]. Then, we compare with Cheng et al. [19] using our dataset on the performance at 0 to 5-days prior to AKI events. Finally, we consider different parameters on the balanced data, different ratio of the (laboratory test, type) pairs and doing prediction, different feature selection methods, the most effective (laboratory test, type) pairs for different prediction times, and the different lengths of data collection window.

### Comparison of existing works

Cheng et al. [19] shows the precision and recall using random forest at 1-day prior to AKI events to be 0.587 and 0.211, respectively. The precision and recall of our method are 0.713 and 0.821 at 1-day prior to AKI events, respectively. In addition, Kate et al. [18] shows that the area under the *receiver operating characteristic* curve, i.e. the AUC using logistic regression is 0.743 when the AKI happens. The AUC of our method is 0.866.

### Comparison of existing methods using our dataset

We implemented the method of Cheng et al. [19] and compared the performance with our approach using our dataset. Table 1 and Table 2 show the performance in terms of precision, recall and F1-score. Although it would be advantageous to predict AKI as early as possible, lengthening the prediction time reduces the performance. The ADQI consensus conference [17] recommended that predicting AKI before 48 to 72 h would give physicians adequate time to modify practice. Our study shows that the F1- score of our method is 0.695 at 2-days prior to AKI events and 0.654 at 3-days prior to AKI events. By using the same dataset, we show a better performance of our approach than Cheng et al. [19].

**Table 1:** The precision and recall of our approach and the three machine learning methods at 0 to 5-days prior to AKI events

Prediction Time	Our approach (Precision/Recall)	Logistic Regression (Precision/Recall)	Random Forest (Precision/Recall)	AdaBoostM1 (Precision/Recall)
AKI Time-0	0.826/0.93	0.779/0.781	0.749/0.742	0.759/0.765
AKI Time-1	0.713/0.821	0.716/0.757	0.687/0.686	0.713/0.734
AKI Time-2	0.64/0.766	0.652/0.646	0.631/0.582	0.639/0.688
AKI Time-3	0.651/0.664	0.633/0.609	0.611/0.567	0.628/0.615
AKI Time-4	0.654/0.668	0.631/0.617	0.603/0.578	0.621/0.651
AKI Time-5	0.596/0.708	0.618/0.602	0.578/0.565	0.606/0.642

**Table 2:** The F1-score of our approach and the three machine learning methods at 0 to 5-days prior to AKI events

Prediction Time	Our approach	Logistic Regression	Random Forest	AdaBoostM1
AKI Time-0	0.875	0.780	0.745	0.762
AKI Time-1	0.759	0.736	0.686	0.723
AKI Time-2	0.695	0.649	0.605	0.662
AKI Time-3	0.654	0.621	0.588	0.621
AKI Time-4	0.659	0.624	0.590	0.635
AKI Time-5	0.646	0.610	0.572	0.623

### Analysis of different parameters

We also compared the performance of using the imbalanced data and balanced data as shown in Table 3. The result of the balanced data shows the average result of the 8 balanced datasets. Although the accuracy of using imbalanced data is higher than the balanced data, the precision, recall, and F1-score are lower than the balanced data. With such an imbalanced dataset, most classifiers will favor the majority class (non-AKI inpatients), resulting in a low accuracy in the minority class (AKI inpatients) prediction. Therefore, we use the balanced data for the following comparisons.

**Table 3:** The prediction performance using imbalanced data and balanced data when AKI happens

	Precision	Recall	F1-score	Accuracy
Balanced data	0.826	0.930	0.875	0.866
Imbalance data	0.6	0.172	0.268	0.901

In data preprocessing, we divided each data set into two parts, one for selecting the (laboratory test, type) pairs and the other for doing prediction. We consider two ratios for these two parts, i.e. 8:2 and 5:5. Table 4 shows that these two ratios have similar performance.

**Table 4:** The F1-score of different ratios for dividing the dataset for selecting the (laboratory test, type) pairs and doing prediction

Prediction Time	8:2 ratio	5:5 ratio
AKI_Time-0	0.875	0.854
AKI_Time-1	0.759	0.752
AKI_Time-2	0.695	0.696
AKI_Time-3	0.654	0.649
AKI_Time-4	0.659	0.66
AKI_Time-5	0.646	0.645

In the (laboratory test, type) pair selection part, we use two methods to select the most effective (laboratory test, type) pairs, including SFS and SBS. Table 5 shows the and F1-score of SFS and SBS at 0 to 5-days prior to AKI events. It shows that SFS has a higher F1-score than SBS for all prediction times.

**Table 5:** The F1-score of SFS and SBS at 0 to 5-days prior to AKI events

Prediction Time	SFS	SBS
AKI_Time-0	0.875	0.740
AKI_Time-1	0.759	0.734
AKI_Time-2	0.695	0.660
AKI_Time-3	0.654	0.594
AKI_Time-4	0.659	0.606
AKI_Time-5	0.648	0.601

In this study, we use the serum creatinine to determine the AKI inpatients and non-AKI inpatients. Therefore, we also compare the performance of only using the serum creatinine and the most effective (laboratory test, type) pairs as shown in Table 6. It shows that the prediction performance of using the most effective (laboratory test, type) pairs is better than only using the serum creatinine at 0 to 5-days prior to AKI events.

**Table 6:** The F1-score of using the most effective (laboratory test, type) pairs and only using the serum creatinine at 0 to 5-days prior to AKI events

Prediction Time	Most Effective Pairs	Serum Creatinine
AKI Time-0	0.875	0.792
AKI Time-1	0.759	0.750
AKI Time-2	0.695	0.541
AKI Time-3	0.654	0.566
AKI Time-4	0.659	0.498
AKI Time-5	0.646	0.509

Table 7 shows the most effective (laboratory test, type) pairs for individual prediction times. The (BUN, Trend) plays more significant role when the prediction time is closer to the AKI time, and the (BUN, Last) is an important pair at 2 to 5-days prior to AKI events. In addition, the most effective (laboratory test, type) pairs for different prediction times are slightly different.

**Table 7:** The most effective (laboratory test, type) pairs for 0 to 5-days prior to AKI events

Prediction Time	Type	Laboratory Test
AKI Time - 0	Trend	Blood Urea Nitrogen (BUN)
AKI Time - 1	Trend	Serum Creatinine
	Trend	Lactic Acid
AKI Time - 2	Last	Blood Urea Nitrogen (BUN)
	Trend	Blood ammonia
AKI Time - 3	Last	Blood Urea Nitrogen (BUN)
	Trend	Blood ammonia
	Trend	Calcium (Ca)
	Trend	Total Protein
AKI Time - 4	Last	Serum Creatinine
	Last	Blood Urea Nitrogen (BUN)
	Trend	Lactic dehydrogenase (LDH)
	Trend	Albumin (ALB)
	Trend	Lactic Acid
	Trend	Phosphorus (P)
AKI Time - 5	Last	Blood Urea Nitrogen (BUN)
	Last	Serum Creatinine
	Trend	Blood ammonia
	Trend	Lactic dehydrogenase (LDH)
	Trend	Calcium (Ca)
	Trend	Phosphorus (P)

In order to relax the requirement for the prediction, we also consider other lengths of the data collection window and show the performance in Table 8. We have the best prediction performance at 2 days prior to AKI events when we set the data collection window to 5 days. Although the performance of the “3 days” is slightly worse than that of the “5 days,” it can predict the AKI for the inpatients with shorter hospital stays.

**Table 8:** The F1-score of different lengths of the data collection window at 0 to 5-days prior to AKI events

Prediction Time	1 day	3 days	5 days
AKI Time-0	0.837	0.868	0.875
AKI Time-1	0.814	0.792	0.759
AKI Time-2	0.609	0.664	0.695
AKI Time-3	0.584	0.669	0.654
AKI Time-4	0.583	0.622	0.659
AKI Time-5	0.523	0.648	0.646

## Discussions

Kate et al. [18] focus on the older inpatients over the age of 60. It uses the demographic information, comorbidity, family history, medication and laboratory test of the inpatients as the predictive variables to predict whether a patient will develop AKI during hospitalization. For each of these variables, only the last recorded value is used. There are two different prediction times in the study, i.e. 24 h after admission and when the AKI happens. The prediction performance always drops after removing a predictive variable. When the laboratory test is removed, the prediction performance drops dramatically when the AKI happens. However, it only incurs a small drop for predicting at 24 h after admission. It means that the laboratory test plays a more significant role when the prediction time is closer to the AKI event. In addition, Kate et al. [26] present a continual prediction model which predicts AKI at the time of a change in the feature values instead of at a particular time. In the training process, the last changed feature value before AKI is used to generate positive examples, and the last changed feature value after AKI is used to generate negative examples. Therefore, it can be used to predict whether an inpatient acquires AKI at any time during the hospitalization. Furthermore, Cheng et al. [19] use the demographic information, laboratory test, vital, medication, and comorbidity as predictive variables to predict the inpatients aged 18–64. For the laboratory test, 14 laboratory tests are selected as the prediction features, and only the last recorded value before the prediction time is used, which is categorized as either “present and normal,” “present and abnormal,” and “unknown” according to standard reference ranges. Three machine learning methods (Logistic Regression [27], Random Forest [28], and AdaboostM1 [29]) are then used with 10-fold cross validation for evaluating the performance. The study predicts the AKI at 0 to 5-days prior to the AKI event and assesses how early and accurately AKI can be predicted. It shows that lengthening the prediction time will reduce the performance.

We compare our method with Kate et al. [18] and Cheng et al. [19] and it shows that our method outperforms the others. Our study is first to consider the last value and the trend of the sequence for each laboratory test. The methods of existing works only use the last recorded value before the AKI event. The trend of the sequence contains more information than the last recorded value, which makes our method perform better.

In addition, we implemented the method of Cheng et al. [19] using our dataset, which also shows our method has a better performance. Cheng et al. [19] shows the precision and recall using random forest at 1-day prior to AKI event to be 0.587 and 0.211, respectively, that is, F1-score = 0.31. The F1-score using our dataset at 1-day prior to AKI events is 0.686. Using our dataset achieves a much better performance. This is because in our dataset we define the exclusion criteria to identify the inpatients who develop AKI during hospitalization and we set the length of the data collection window to 5 days.

Finally, we individually select the most effective (laboratory test, type) pairs to do the prediction for different days of early prediction. The existing works select the fixed laboratory tests for different days prior to AKI. Therefore, we can have the better performance.

## Conclusions

AKI is a common clinical event among inpatients and it can result in significant mortality, especially for older inpatients. Early identification of the high-risk older inpatients to prevent them from acquiring AKI is therefore important. In this study, we proposed an approach to early predict AKI, which shows a better performance compared with the existing works. In addition, we found that the earlier the AKI is predicted, the more (laboratory test, type) pairs are required, and the BUN is an important laboratory test in the prediction. However, more studies are needed to determine if early prediction of AKI decreases the development of AKI and decreases the AKI associated adverse outcomes.

In the future, we will consider to incorporate other data types such as demographic information, comorbidities, family history, and medications to increase our prediction performance. Furthermore, we will extend this approach and develop a system for early prediction of other major complications to help better disease management for inpatients.

## Data Availability

The dataset can only be used after getting the approval from China Medical University Hospital.

## Abbreviations

ADQI: Acute Dialysis Quality Initiative
AKI: Acute Kidney Injury
AKIN: Acute Kidney Injury Network
ALB: Albumin
BUN: Blood Urea Nitrogen
Ca: Calcium
CCR: Creatinine Clearance Rate
CCR: Creatinine Clearance Rate
CK-MB: Creatine kinase-MB
DTW: Dynamic time warping
EHR: Electronic health records
KNN: K-nearest neighbor
LDH: Lactic dehydrogenase
LS: Similarity score of the last value
P: Phosphorus
SBS: Sequential backward selection
SCr: Serum Creatinine
SFS: Sequential forward selection
SGPT: Serum Glutamic-Pyruvic Transaminase
TS: Similarity score of the trend
